# Immune Protection Induced on Day 10 Following Administration of the 2009 A/H1N1 Pandemic Influenza Vaccine

**DOI:** 10.1371/journal.pone.0014270

**Published:** 2010-12-09

**Authors:** Yizhuo Sun, Chao Bian, Ke Xu, Weibin Hu, Tongyan Wang, Jun Cui, Hongqiang Wu, Zhiyang Ling, Yongyong Ji, Guomei Lin, Lin Tian, Yanyan Zhou, Bingnan Li, Guiyu Hu, Ning Yu, Wenqi An, Ruowen Pan, Paul Zhou, Qibin Leng, Zhong Huang, Xiaowei Ma, Bing Sun

**Affiliations:** 1 Key Laboratory of Molecular Virology and Immunology, Institut Pasteur of Shanghai, Chinese Academy of Sciences, Shanghai, China; 2 Key Laboratory of Molecular Cell Biology, Institute of Biochemistry and Cell Biology, Shanghai Institutes for Biological Sciences, Chinese Academy of Sciences, Shanghai, China; 3 Shanghai Immune Biotech Company, Shanghai, China; 4 Hualan Biological Bacterin Company, Xinxiang, China; Southern Illinois University School of Medicine, United States of America

## Abstract

**Background:**

The 2009 swine-origin influenza virus (S-OIV) H1N1 pandemic has caused more than 18,000 deaths worldwide. Vaccines against the 2009 A/H1N1 influenza virus are useful for preventing infection and controlling the pandemic. The kinetics of the immune response following vaccination with the 2009 A/H1N1 influenza vaccine need further investigation.

**Methodology/Principal Findings:**

58 volunteers were vaccinated with a 2009 A/H1N1 pandemic influenza monovalent split-virus vaccine (15 µg, single-dose). The sera were collected before Day 0 (pre-vaccination) and on Days 3, 5, 10, 14, 21, 30, 45 and 60 post vaccination. Specific antibody responses induced by the vaccination were analyzed using hemagglutination inhibition (HI) assay and enzyme-linked immunosorbent assay (ELISA). After administration of the 2009 A/H1N1 influenza vaccine, specific and protective antibody response with a major subtype of IgG was sufficiently developed as early as Day 10 (seroprotection rate: 93%). This specific antibody response could maintain for at least 60 days without significant reduction. Antibody response induced by the 2009 A/H1N1 influenza vaccine could not render protection against seasonal H1N1 influenza (seroconversion rate: 3% on Day 21). However, volunteers with higher pre-existing seasonal influenza antibody levels (pre-vaccination HI titer ≥1∶40, Group 1) more easily developed a strong antibody protection effect against the 2009 A/H1N1 influenza vaccine as compared with those showing lower pre-existing seasonal influenza antibody levels (pre-vaccination HI titer <1∶40, Group 2). The titer of the specific antibody against the 2009 A/H1N1 influenza was much higher in Group 1 (geometric mean titer: 146 on Day 21) than that in Group 2 (geometric mean titer: 70 on Day 21).

**Conclusions/Significance:**

Recipients could gain sufficient protection as early as 10 days after vaccine administration. The protection could last at least 60 days. Individuals with a stronger pre-existing seasonal influenza antibody response may have a relatively higher potential for developing a stronger humoral immune response after vaccination with the 2009 A/H1N1 pandemic influenza vaccine.

## Introduction

From April 2009, a novel influenza virus strain emerged and quickly spread from the United States and Mexico to the rest of the world[1]. This 2009 A/H1N1 pandemic caused more than 18,000 deaths worldwide[2]. Great attention and effort were applied to conquer this pandemic[3]. Recently the WHO declared our entry into the post-pandemic period and expected the H1N1 pandemic virus to take on the behavior of a seasonal influenza virus, which may continue to circulate for some years to come[4].

This 2009 A/H1N1 influenza virus is of swine origin and its unique genome is a combination of both American and Eurasian lineages[5], [6]. The antigenicity of the virus is distinct from that of the seasonal human H1N1 influenza A virus and people, especially young people, generally lack immune protection against this new virus[5], [6].

Several clinical trials have revealed that the split-virus vaccine against the 2009 A/H1N1 virus, either with or without adjuvant, is very effective and can establish sufficient protection in the general population[7], [8], [9], [10]. From these studies, we have determined that 14 or 21 days after vaccine administration, the volunteers generally develop protection against the 2009 A/H1N1 influenza virus. However, our knowledge regarding the dynamic, changing profile of the antibody response at different time points after vaccine administration is still limited. Here we conducted a single-dose administration of a non-adjuvanted 2009 A/H1N1 influenza monovalent split-virus vaccine in a group of 58 volunteers and studied the specific antibody response before and at days 3, 5, 10, 14, 21, 30, 45 and 60 after vaccine administration. We found that at as early as day 10, most of the volunteers developed a protective antibody response (mainly IgG) against the 2009 A/H1N1 influenza virus. This specific response can last at least 60 days without significant reduction.

Another important question that draws much attention is whether the pre-existing seasonal influenza antibody response plays a role during the 2009 A/H1N1 influenza vaccine administration. Some studies have suggested that the recent seasonal influenza vaccine administration is unlikely to provide protection against the 2009 A/H1N1 pandemic influenza infection[11], [12]. In this study, we observed that people with a higher seasonal H1N1 influenza antibody background more easily develop a stronger antibody response against the 2009 A/H1N1 influenza after vaccine administration. Similar to this human study observation, an animal experiment also showed that mice pre-immunized with seasonal influenza vaccine yielded a slightly higher GMT after the 2009 A/H1N1 influenza vaccination.

These results indicate that the pre-existing immune status with respect to the seasonal influenza could be an indicator for assessing the immune response against the 2009 A/H1N1 pandemic influenza after vaccination.

## Methods

### Ethics and Subjects

This study was approved by the Institutional Review Board of Institut Pasteur of Shanghai, CAS. Healthy non-pregnant adults between the ages of 18 and 60 were eligible for enrollment. Subjects who experienced the pandemic 2009 A/H1N1 influenza infection or vaccine administration were excluded by carefully reviewing the influenza related clinical records or history. All the subjects were from Xinxiang, Henan province of China. Written informed consent that indicated the volunteers' complete understanding of the experimental procedure was obtained from all subjects. Fifty eight subjects including 46 men and 12 women were recruited for the study. The ages of the volunteers ranged from 19 to 53 with an average age of 28 and median of 26.

Mice used in this study were handled in strict accordance with the Guidelines for Animal Care and Use of the Institut Pasteur of Shanghai, Chinese Academy of Sciences. The protocol was approved by the Institutional Committee for Animal Experiments of the Institut Pasteur of Shanghai, CAS. All surgery was performed under ether anesthesia. All efforts were made to minimize suffering.

### Vaccine

The monovalent 2009 A/H1N1 influenza split-virus vaccine without adjuvant was produced by Hualan Biological Bacterin Company, China. It was prepared from reassortant strain X-179A (by New York Medical College), was derived from the A/California/7/2009 (H1N1) virus and was recommended by the World Health Organization. It has been demonstrated effective and safe for use[8], [9]. In this study, we also used another seasonal H1N1 influenza vaccine for hemagglutination inhibition (HI) assay. It was prepared from reassortant strain IVR-148 and with HA gene from the A/Brisbane/59/2007 (H1N1) strain. This seasonal influenza virus vaccine was also produced by Hualan Biological Bacterin Company.

### Study procedures

The trial lasted from October to December, 2009. The subjects received one dose of 15 µg split-virus vaccine against the 2009 A/H1N1 influenza virus on day 0. Serum samples were collected from all subjects before vaccination and after vaccination on day 3, day 5, day 10, day 14, day 21, day 30, day 45 and day 60. The serum samples collected before day 30 were complete and on days 30, 45 and 60 at least 51 samples were collected for each time point for analysis. The influence of variation of the sample number was considered and excluded in statistical analysis with the software used.

### Laboratory assays

Antibody titers of all serum samples were determined by means of hemagglutination inhibition (HI) assay according to established procedures and with chicken erythrocytes[8]. Briefly, serum samples were treated with receptor-destroying enzyme (RDE, cholera filtrate) at 37°C for 18 hours, and then heated at 56°C for 30 minutes. In our HI assay, serum samples were diluted from 1∶10 to 1∶5120 and we recorded as the HI titer the highest dilution rate that caused complete hemagglutination inhibition against 4 hemagglutinating units (HAU) of virus. Every serum sample was assayed in two independent HI tests against the formaldehyde inactivated 2009 A/H1N1 influenza virus (X-179A reassortant strain) and the seasonal H1N1 influenza virus (IVR-148 reassortant strain). All tests were repeated at least twice for recheck of the authenticity of the data.

An enzyme-linked immunosorbent assay (ELISA) was also performed briefly as the following procedure. Ninety-six-well micro-titer plates (Nunc) were coated with insect cell expressed hemagglutinin (HA) protein in 0.1 M carbonate buffer (pH 9.6) at a concentration of 10 µg/ml at 4°C overnight. Blocking buffer and dilution buffer consisted of phosphate buffered saline (PBS) containing 10% bovine serum and 0.1% Tween-20. After blocking of the plates at 37°C for 2 hours, the plates were washed and 1∶50 diluted sera added, then incubated at 37°C for 2 hours. After washing to remove non-specific binding antibodies, horse radish peroxidase conjugated goat anti-human IgG or IgM antibody (Sigma) was diluted and added to the plates which were incubated at 37°C for another hour. Tetramethylbenzidine (TMB) was used as the substrate and the absorbance was measured at 450 nm with a micro-plate autoreader (Thermo).

### Mice Vaccination

Six-week-old female BALB/c mice were purchased from Shanghai Laboratory Animal Center, Chinese Academy of Sciences and were kept in specific pathogen free (SPF) facilities. Mice were divided into two groups: Group A and Group B (each group, n = 10). On day 0, mice in Group A were vaccinated with PBS (control) and those in Group B were vaccinated with the 2008-09 trivalent seasonal influenza vaccine. Twenty one days after the first vaccination (on day 21), both groups were vaccinated with the 2009 A/H1N1 influenza monovalent split-virus vaccine. Sera were collected on day 0, 21 and 35. On each time point, the serum HI titers against both the pandemic and the seasonal influenza vaccines were determined by the hemagglutination inhibition assay as described above.

Both of the 2009 A/H1N1 influenza monovalent split-virus vaccine and the 2008-09 trivalent seasonal influenza vaccine were produced by Hualan Biological Bacterin Company, China. 6 µg seasonal influenza vaccine or 7.5 µg 2009 A/H1N1 pandemic influenza vaccine (in 150 µl sterile PBS, pH 7.4) per mouse was injected from the quadriceps muscles of both legs using a 29-gauge needle (BD) with each leg receiving half of the vaccine administrated. Mouse blood was collected using the retro-orbital bleeding method and sera were obtained by centrifugation (10,000 g, 10 min) to remove the blood clot after incubation at 37°C for 2 hours and then at 4°C overnight.

### Statistical methods

In this study, the immunogenicity endpoints were seroprotection rate, seroconversion rate and geometric mean titer (GMT) according to the international guidelines established by the European Committee for Medicinal Products to evaluate influenza vaccines[13]. Based on the criteria established for seasonal influenza, seroprotection was defined as an HI titer of 1∶40 or more. Seroconversion was defined as an increase of the HI titer by a factor of four or more with the postvaccination HI titer more than 1∶40. HI titer below 1∶10 was assigned a value of 1∶5 in calculating the GMT.

The Student's t-test and the Pearson's chi-squared test were used to compare different groups of data. P values of 0.05 or less were considered significant. All statistical data were calculated using R software (version 2.10.1).

## Results

### Dynamics of antibody response to 2009 A/H1N1 vaccine in human volunteers

**Figure 1:**
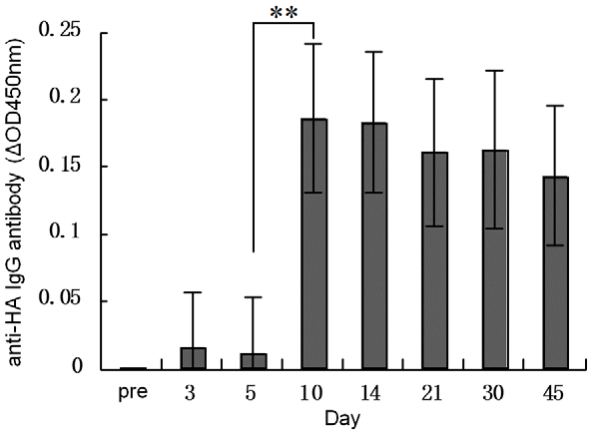
ELISA to detect specific anti-HA IgG antibody at different time points in the sera of volunteers for the 2009 A/H1N1 influenza vaccine administration. The OD450 nm on different days were measured and results were displayed as ΔOD450 nm with each prevaccination (baseline) OD subtracted in every subject's serial samples. "pre": pre-vaccination. The OD value of the pre-vaccination sera was 0.76±0.06 (IgG baseline). The blank OD (no sera added) of the ELISA was 0.05. **: p<0.001, two-way Student's t-test.

Serum levels of antibody against hemagglutinin were determined using a hemagglutination inhibition assay, which is a standard assay used in the process of licensing influenza vaccines. Seroprotection rate, seroconversion rate and geometric mean titer are calculated for the HI assay to express the sero-response after influenza virus infection or vaccination. Table 1 shows that before vaccination the seroprotection rate and GMT against 2009 A/H1N1 virus were kept at a baseline, 12% and 16 respectively. After vaccination, they increased gradually. On day 5, seroprotection rate, seroconversion rate and GMT achieved 47%, 19% and 32 respectively. On day 10, seroprotection rate, seroconversion rate and GMT achieved 93%, 72% and 86, which levels are almost sufficient to protect subjects against the 2009 A/H1N1 virus infection. The explosive growth of the antibody titer occurred between day 5 and day 10 (p = 7.0e-10). A reasonable explanation is that specific IgG antibodies against HA began to be generated at this stage (Figure 1). On day 14, seroprotection rate, seroconversion rate and GMT achieved 97%, 86%, and 112 respectively. The antibody titer almost reached its maximum on day 14 and remained nearly unchanged from day 14 to day 60. We concluded that the 2009 A/H1N1 influenza vaccine was effective in establishing protection on day 10 after vaccination and that this protection could be maintained for at least 60 days. Compared with the baseline, seroprotection rate, seroconversion rate and GMT on day 5 and thereafter all showed very big statistical differences with the p values generally less than 0.001 (except the seroconversion rate on day 5). The pattern of the HA-specific IgG antibody response determined by ELISA was almost in accordance with the HI assay results (Figure 1). The specific IgM response against HA is very low and did not show significant differences among different time points in our ELISA experiments (data not shown). There was no cross reactivity of the IgG and IgM ELISAs under the working dilution rates of the goat anti-human IgG or IgM enzyme conjugated antibody, a finding that was determined with a direct ELISA using human IgG and IgM protein for detection (data not shown). A dilution rate of 1∶200 or less of the sera could show a positive result in the ELISA and 1∶50 was chosen to obtain an appropriate OD value.

**Table 1:** Dynamic Changes of the Hemagglutination-Inhibition Titers against the 2009 A/H1N1 Virus after Administration of the 2009 A/H1N1 Influenza Vaccine.

Time	Seroprotection rate -% (95% CI)	Seroconversion rate - % (95% CI)	GMT (95% CI)
Pre (N = 58)	12 (5-23)	-	16 (13-20)
day 3 (N = 58)	19 (10-31)	5 (1-14)	19 (15-24)
day 5 (N = 58)	47 (33-60)**	19 (10-31)*	32 (27-38)**
day 10 (N = 58)	93 (83-98)**	72 (59-83)**	86 (68-108)**
day 14 (N = 58)	97 (88-100)**	86 (75-94)**	112 (88-142)**
day 21 (N = 58)	97 (88-100)**	86 (75-94)**	113 (89-144)**
day 30 (N = 51)	98 (90-100)**	88 (76-96)**	117 (92-149)**
day 45 (N = 55)	95 (85-99)**	84 (71-92)**	117 (91-150)**
day 60 (N = 56)	95 (85-99)**	84 (72-92)**	113 (88-145)**

Seroprotection is defined as HI titer of 1∶40 or more. Seroconversion is defined as postvaccination HI titer of 1∶40 or more, increasing at least four times. GMT = geometric mean titer. HI titers below 1∶10 were assigned a value of 1∶5, in calculating the geometric mean titer.

*(p<0.05) and

**(p<0.001) represent the statistical significances compared with the pre-vaccination values (day 3 value for seroconversion rate). The Pearson's chi-squared test was used for the seroprotection and seroconversion rate analysis, the two-way Student's t-test for the GMT analysis. Pre: pre-vaccination.

We also analyzed the dynamic changes of the antibody response against seasonal H1N1 virus after vaccination with the 2009 A/H1N1 influenza vaccine (Table 2). Before vaccination, the seroprotection rate and GMT against seasonal H1N1 virus were 59% and 30 respectively. On day 21, the seroprotection rate and GMT were 64% and 37, which barely increased as compared with the baseline (p = 0.70). Obviously, the 2009 A/H1N1 influenza vaccine showed no cross protection against the seasonal H1N1 influenza virus. Similar results were also obtained from HI experiments concerning a 2008-09 trivalent seasonal influenza vaccine (data not shown).

**Table 2:** Dynamic Changes of the Hemagglutination-Inhibition Titers against the Seasonal H1N1 Virus after Administration of the 2009 A/H1N1 Influenza Vaccine.

Time	Seroprotection rate -% (95% CI)	Seroconversion rate - % (95% CI)	GMT (95% CI)
Pre (N = 58)	59 (45-71)	-	30 (24-38)
day 3 (N = 58)	59 (45-71)	3 (0-12)	32 (25-40)
day 5 (N = 58)	59 (45-71)	3 (0-12)	32 (25-41)
day 10 (N = 58)	60 (47-73)	3 (0-12)	36 (29-46)
day 14 (N = 58)	64 (50-76)	3 (0-12)	38 (30-47)
day 21 (N = 58)	64 (50-76)	3 (0-12)	37 (30-46)

### Pre-existing antibody to seasonal influenza is predictive of a higher antibody response to the 2009 A/H1N1 vaccine

To investigate the relationship of the antibody response of the seasonal influenza and the 2009 A/H1N1 influenza, the 58 subjects were further divided into two groups based upon their baseline HI titers against the 2008-09 seasonal trivalent influenza vaccine. Group 1 contained 38 subjects whose baseline HI titers against seasonal influenza vaccine were ≥1∶40. Group 2 contained 20 subjects whose seasonal influenza HI titers were less than 1∶40. The seroprotection rate, seroconversion rate and GMT for the 2009 A/H1N1 influenza vaccine in Group 1 and Group 2 were calculated at each time point until day 21 post vaccination (Table 3). There was no statistical difference between Group 1 and Group 2 with regard to seroprotection rate and seroconversion rate (p = 1.0 on day 21, data not shown). However, Group 1 achieved a relatively higher GMT (146 on day 21 after vaccination), in contrast to Group 2 which generated a relatively lower one (70 on day 21). Group 1 showed markedly higher antibody titers than Group 2 at each time point after vaccination (p<0.05), especially on day 10, 14 and day 21 (p<0.001).

**Table 3:** Geometric Mean HI Titers against the 2009 A/H1N1 Influenza Virus in Two Groups of People with Different Seasonal Influenza Antibody Levels.

	Seroprotective for seasonal influenza prior to vaccination?	
Time	Yes† (N = 38) (95% CI)	No ° (N = 20) (95% CI)
Pre (N = 58)	18 (14-24)*	12 (9-16)
day 3 (N = 58)	23 (17-31)*	14 (11-18)
day 5 (N = 58)	37 (29-48)*	25 (21-29)
day 10 (N = 58)	111 (82-150)**	53 (40-69)
day 14 (N = 58)	143 (104-198)**	70 (54-89)
day 21 (N = 58)	146 (105-203)**	70 (54-89)

† Individuals with prevaccination seasonal influenza HI titer ≥1∶40.

° Individuals with prevaccination seasonal influenza HI titer <1∶40.

*(p<0.05) and

**(p<0.001) represent the statistical differences between the two groups, two-way Student's t-test.

### Mouse correlate of Table 3

To further confirm the observation in Table 3, a mouse experiment was conducted. On day 0, two groups (10 in each) of six-week-old SPF BALB/c mice were vaccinated with PBS (Group A) or the seasonal influenza vaccine (Group B) respectively. Twenty one days post vaccination (on day 21), both groups were further vaccinated with the 2009 A/H1N1 influenza vaccine. Sera were collected on day 0, 21 and 35. The GMT against the seasonal and the 2009 A/H1N1 influenza vaccine were determined and compared with each other. As shown in Table 4, the significant higher HI titers against the seasonal influenza vaccine on day 21 and 35 in Group B as compared to Group A (p<0.001) demonstrated a successful seasonal influenza vaccination. The strong antibody response against the 2009 A/H1N1 influenza vaccine on day 35 in both groups indicated an effective administration of the 2009 A/H1N1 influenza vaccine. Importantly, the GMT against the 2009 A/H1N1 influenza vaccine on day 35 of Group B was slightly higher than that of Group A (197 to 149). Although the difference is not statistical significant (p = 0.17), the result is in accordance with the observation of the human study (Table 3). This result suggests that the pre-existing immune response background of the seasonal influenza may be helpful for generating a stronger antibody response against the 2009 A/H1N1 influenza vaccine.

**Table 4:** Geometric Mean HI Titer against the Seasonal and the 2009 A/H1N1 Influenza Virus in Two Groups of Mice with Different Vaccination Procedures#.

	Day 0		Day 21		Day 35	
GMT (95% CI)	Seasonal	2009 A/H1N1	Seasonal	2009 A/H1N1	Seasonal	2009 A/H1N1
Group A (N = 10)	5 (5-5)	5 (5-5)	5 (5-5)	5 (5-5)	5 (5-5)	149 (104-215)
Group B (N = 10)	5 (5-5)	5 (5-5)	160 (115-223)**	5 (5-5)	160 (115-223)**	197 (155-250)

# Group A: mice immunized with PBS (control) on day 0 and the 2009 A/H1N1 influenza vaccine on day 21; Group B: mice immunized with the 2008-09 trivalent seasonal influenza vaccine on day 0 and the 2009 A/H1N1 influenza vaccine on day 21.

Seasonal: geometric mean HI titer against the 2008-09 trivalent seasonal influenza vaccine; 2009 A/H1N1: geometric mean HI titer against the 2009 A/H1N1 pandemic influenza vaccine.

**(p<0.001) represents the statistical differences between Group A and Group B (two-way Student's t-test).

## Discussion

Influenza A viruses cause epidemics every year and occasionally global pandemics due to their highly variable genome. Seasonal influenza vaccines are revised every 1–3 years to cope with mutations in the HA and NA proteins of circulating viruses[14]. However, the mechanism by which a new pandemic influenza virus appears and the strategy applied by the virus to escape human immunity are unclear. Vaccine development is, to date, the best way to prevent influenza virus infection.

To determine the antibody's dynamic, changing profile following the 2009 A/H1N1 influenza vaccine administration is a very important task. It could allow prediction of the point at which the influenza vaccine takes effect and how long the effect will last. Our work demonstrates that most vaccine recipients obtain protection as early as 10 days after vaccination and that protection is maintained for at least 60 days. This protection is likely due to the anti-HA IgG subtype antibodies.

Importantly, we observed that people with a better seasonal influenza antibody response more easily reached a higher protective antibody level after the 2009 A/H1N1 influenza vaccine administration. This may be due to the fact that some people may have a good immune response and in spite of seasonal influenza or the 2009 A/H1N1 pandemic influenza they encounter, a generally more intensive antibody response could be induced among them. In addition, other viral infections may also contribute to the low antibody response against the seasonal influenza. Another explanation is that the pre-existing seasonal influenza immune response provides some priming effect for the 2009 A/H1N1 influenza vaccine administration. We noticed that Del Giudice et al. carried out experiments in ferrets and demonstrated that seasonal influenza vaccination could provide a priming effect to the 2009 A/H1N1 influenza vaccine[15]. Garcia-Garcia et al. also demonstrated clinically that seasonal trivalent influenza vaccine could partially protect people from the 2009 A/H1N1 influenza infection[16]. As indicated by these reports, the stronger antibody response in the higher seasonal influenza antibody level group, as we observed, might also be due to the priming effect of the pre-existing seasonal influenza immune response, an assumption also supported by the observation that serum IgG against HA increased dramatically between day 5 and day 10 after vaccination (Figure 1).

The result of the mice experiment is similar to that of the human study though not dramatic, which might be due to the small animal number. It indicates that the priming effect of the pre-existing seasonal influenza immune response may have some promoting role on the pandemic influenza vaccination. However, in the human study, we cannot neglect the influence of the subject's immune system on generating immune response against the vaccine. Both of the priming effect and the immune system status could be important factors influencing the protection result in human vaccination.

The reason for using 1∶40 as the criterion to define Group 1 and Group 2 in Table 3 is that this titer is considered seroprotective by a number of authorities[13], [17]. Work published in 1984 showed that a titer of 1∶42 for H1N1 and 1∶44 for H3N2 provided 50% protection against infection[18].

Our study suggests that to obtain an optimal effect for the 2009 A/H1N1 influenza vaccination, one should consider the recipient's previous seasonal influenza antibody level. It is a potential indicator for predicting whether a good vaccination effect can be achieved. Our results suggest that multiple immunizations may be required for people with low seasonal influenza antibody levels.
