# Expression of Concern: Immune Protection Induced on Day 10 Following Administration of the 2009 A/H1N1 Pandemic Influenza Vaccine

**DOI:** 10.1371/journal.pone.0039314

**Published:** 2012-05-25

**Authors:** The PLoS ONE Editors

Following the publication of the article “Immune Protection Induced on Day 10 Following Administration of the 2009 A/H1N1 Pandemic Influenza Vaccine”, the Editors of *PLoS ONE* noticed that the article described findings from a clinical trial. In line with *PLoS ONE’s* editorial policies for clinical trials; the article should have included a trial registration number for the study and a copy of the trial protocol.

The Editors followed up on this matter with the authors, who indicated that the trial had not been registered at a public registry. The Editors were also concerned about the documentation and clarifications provided by the authors in response to the request for the study protocol and details on ethical approval for the study. In the light of the concerns raised in relation to the article, the Editors contacted the Institut Pasteur of Shanghai to request an institutional investigation into the research reported in the article.

The Institut Pasteur of Shanghai has now completed their institutional investigation. While the investigation has not questioned the results reported in the article, the enquiry has revealed concerns about some aspects of the study. In the light of the findings of this enquiry, the Editors issue this expression of concern to make readers aware of the following concerns in relation to this trial:

- The article reports that the clinical component of the study was approved by the Institutional Review Board of Institut Pasteur of Shanghai. However, the investigation committee has stated that the clinical study was in fact approved by the Henan CDC Ethics Committee.
- The Editors feel that the reporting of the clinical trial in the article is inadequate given that no information is provided regarding the setting for recruitment of the participants. The investigation committee has reported that the participants in the study were employees of Hualan Biological Bacterin Company, China, which is one of the funders of the study, the manufacturer of the vaccine tested, and the employer of several of the authors.
- The article reports that 58 subjects were recruited for the study, however, the institutional investigation established that approximately 130 volunteers were recruited, while only 58 completed the full protocol.
- As outlined above, the clinical study was not registered as a clinical trial. *PLoS ONE* supports the position of the International Committee of Medical Journal Editors (ICMJE) on trial registration and follows the WHO definition of a clinical trial, which constitutes : *“...any research study that prospectively assigns human participants or groups of humans to one or more health-related interventions to evaluate the effects on health outcomes.*” In this study participants were prospectively recruited, received a vaccine against the 2009 A/H1N1 influenza virus and their immune response was evaluated following vaccination. We consider the vaccination to be health-related intervention and the measurement of the immune response to constitute a health outcome, and as a result, the Editors consider that the study fulfills the criteria for a clinical trial and should have been prospectively registered at a public repository.

